# Sheep tail fat inhibits the proliferation of non-small-cell lung cancer cells *in vitro* and *in vivo*

**DOI:** 10.3389/fphar.2022.917513

**Published:** 2022-08-11

**Authors:** Changzhi Xu, Lanlan Zhang, Huimin He, Xiaoyi Liu, Xinxin Pei, Tengfei Ma, Bingbing Ma, Wenchu Lin, Buchang Zhang

**Affiliations:** ^1^ Institutes of Physical Science and Information Technology, Anhui University, Hefei, Anhui, China; ^2^ Anhui Tianxiang Grain and Oil Food Co., Ltd., Fuyang, Anhui, China; ^3^ Fuyang Tianxiang Food Technology Co., Ltd., Fuyang, Anhui, China; ^4^ High Magnetic Field Laboratory, Chinese Academy of Sciences, Hefei, Anhui, China

**Keywords:** sheep tail fat, non-small-cell lung cancer, heptadecanoic acid, antiproliferative effect, Akt/S6K signaling pathway

## Abstract

Increasing evidence suggests that numerous edible oils may function as adjuvant dietary therapies to treat cancer. We previously reported that the odd-chain saturated fatty acid (OCSFA), heptadecanoic acid (C17:0), profoundly inhibits non-small-cell lung cancer (NSCLC) cell proliferation. However, the antitumor potential of edible lipids rich in C17:0 remains unclear. Here, we determined that sheep tail fat (STF) is a dietary lipid rich in C17:0 and exhibited the greatest inhibitory effect against three NSCLC cell lines (A549, PC-9, and PC-9/GR) among common dietary lipids. Cell migration experiments demonstrated that STF could significantly inhibit the wound healing capacity of three NSCLC cell lines by promoting the generation of reactive oxygen species (ROS) and subsequent cell death. Mechanistic studies showed that STF suppressed NSCLC cell growth by downregulating the Akt/S6K signaling pathway. Furthermore, administration of STF reduced tumor growth, weight, and expression of the proliferative marker Ki-67 in nude mice bearing A549 xenografts. Collectively, our data show that STF has antitumor activity against NSCLC, implying that dietary intake of C17:0-rich STF may be a potential adjuvant therapy for NSCLC.

## Introduction

Lung carcinoma is a fatal disease prevalent worldwide (Siegel et al., 2022). Patients with non-small-cell lung cancer (NSCLC), the most prevalent subtype of lung cancer, often have a poor 5-year survival rate, mainly due to acquired resistance to therapeutic drugs, such as human epidermal growth factor receptor-tyrosine kinase inhibitors (EGFR-TKIs). Studies have demonstrated that several mechanisms may contribute to drug resistance in lung cancer (Lin et al., 2014). Therefore, novel therapies are urgently required to synergistically treat lung cancer.

Dietary therapy is currently considered a useful adjuvant approach for treating various diseases, including a range of cancers (Tajan and Vousden, 2020). Numerous studies have shown that the composition and mode of food we consume can function as an intervention for a range of cancers. As an essential nutrient, edible lipids can provide energy and membrane components and act as a signaling molecule in the regulation of key cellular properties. Dietary lipids also play an important role in cancer development. Abnormal lipid metabolism has been shown to be a key characteristic of cancer cells, and approaches targeting lipid metabolism have shown promise for cancer therapy (Bian et al., 2021). Recently, lipids extracted from plants and deep-sea fish, including walnut oil (Batirel et al., 2018), volatile oils from *Alpinia officinarum* Hance (Zingiberaceae) (Li et al., 2018), *Phlomis aurea* Dence (Lamiaceae) (Torky et al., 2021), and fish oil (Dierge et al., 2021), have been shown to exert antitumor effects on various cancer cells *in vitro* and *in vivo*. The activity of these oils is thought to be due to their high content of unsaturated fatty acids (UFAs), such as docosahexaenoic acid (DHA) and eicosapentaenoic acid (EPA) in fish oil. These UFAs exert cytotoxicity toward cancer cells through different mechanisms. For instance, DHA and EPA were recently reported to inhibit cancer cells by triggering ferroptosis (Dierge et al., 2021). However, lipids rich in saturated fatty acids (SFAs) have rarely been reported to repress tumor growth.

Heptadecanoic acid (C17:0), also known as margaric acid, is a representative odd-chain saturated fatty acid (OCSFA) in ruminants. Studies have shown that C17:0 intake is inversely associated with coronary heart disease (Khaw et al., 2012), type 2 diabetes (Meikle et al., 2013), and multiple sclerosis (Holman et al., 1989). We recently reported that C17:0 displayed a significant anti-proliferative effect and promoted the inhibitory effect of the EGFR TKI gefitinib in NSCLC cells (Xu et al., 2019). Therefore, foods rich in C17:0 may have the potential for use in the context of lung cancer treatment. A number of foods/organisms, including ruminant lipids or milk fat (Jenkins et al., 2015), ripe pulp in dessert banana (Ningsih et al., 2021) and certain microalgae [such as *Messatrum gracile* (Chlorophyceae) (Divya Kuravi and Venkata Mohan, 2021)], contain high levels of C17:0. Among these sources, C17:0 can most readily be obtained from dietary ruminant lipids. Here, we determined that C17:0 is abundant in sheep tail fat (STF). STF has been widely used in the diet and food industries, such as halal food, and the confectionery industry (Aksu, 2009). It is frequently used in traditional Uyghur and Kazakh medicine in China as a natural entity to treat a range of diseases, such as colds in children, rheumatism, and constipation (Kulxax, 2011). Therefore, STF is a functional food resource that can be beneficial for human health.

In this study, we hypothesized that STF might effectively suppress the proliferation in NSCLC cells. Specifically, the potential growth inhibitory, pro-apoptotic, and anti-migration capacities of STF were examined. The *in vivo* antitumor effects of STF were also investigated.

## Materials and methods

### Preparation of sheep tail fat

STF and other ruminant lipids (including beef tallow, perirenal fat, omentum fat, and suet fat from sheep) supplied by Anhui Tianxiang grain and oil Food, Ltd. were purchased from Xinjiang Uygur Autonomous Region or the Inner Mongolia Autonomous Region. STF was extracted by heating, filtered with gauze, and stored separately at 4°C. Other common dietary lipids, including lard, olive oil, peanut oil, and sunflower oil, were purchased from domestic edible oil manufacturers in China.

### Fatty acid profiles by gas chromatography-mass spectrometry analysis

STF (0.06 g) and the other lipids were dissolved in 8 ml of n-hexane. Two milliliters of KOH-CH_3_OH buffer was added and mixed, followed by incubation for 3 min. The supernatant was transferred to a sampling tube and subjected to gas chromatography-mass spectrometry (GC-MS) analysis, as previously described (Xu et al., 2019). The fatty acid content was quantified using mixed standards. The fatty acid compositions of STF and the other lipids (Zheng et al., 2014; Gao et al., 2017; Zhang et al., 2021; Zhao et al., 2021; Luo et al., 2022) are presented in Supplementary Table S1.

### Cell culture

The cancer cell lines were stored in our laboratory. All cells [A549, PC-9, and PC-9 acquired gefitinib-resistance (PC-9/GR) cells] were cultured as previously described (Fei et al., 2013; Xu et al., 2019).

### Cell proliferation assay

All cells tested were seeded at equal densities in 96-well plates at 37°C overnight. Cells were incubated with serum-free medium for 12 h. Fresh medium containing varying concentrations of STF (0, 1, or 2 g/l) was added, and the cells were cultured for another 24 h. STF was dissolved in DMSO and diluted in the culture medium (Batirel et al., 2018). A final concentration of DMSO below 0.1% was shown to exert no cytotoxicity. 3-(4,5-dimethylthiahiazo-2-yl)-3,5-diphenyltetrazolium bromide (MTT) was added for 4 h at 37°C, and the absorption at 490 nm was determined (Xu et al., 2019).

### Colony formation assay

NSCLC cells (200–300) were plated overnight in 6-well plates. The cells were switched to medium without fetal bovine serum (FBS) for 6 h, followed by the administration of an STF-containing culture medium every 48 h. Two weeks later, the number of cell colonies was counted, as previously described (Xu et al., 2019).

### Flow cytometry assay of reactive oxygen species production and apoptosis

To estimate the reactive oxygen species (ROS) production, equal numbers of cells with the indicated treatments were washed thrice with ice-cold PBS and fixed with ice-cold 75% ethanol at 4°C overnight. The samples were resuspended in 500 μl of ice-cold PBS and incubated with 20 μl of RNase A solution at 37°C for 30 min, followed by filtering and staining with 2′-7′dichlorofluorescin diacetate (DCFH-DA) dye at 4°C for 1 h. The samples were then analyzed by flow cytometry (Gallios™, Beckman). Similarly, cell samples with the indicated treatments were stained with Annexin V-fluorescein isothiocyanate/propidium iodide (FITC/PI) according to the manufacturer’s protocol to assay cell death and apoptosis.

### Immunoblotting

Cells were collected and subjected to immunoblotting, as previously described (Xu et al., 2019). Primary antibodies against glucose transporter 1 (GLUT1), p-Akt, p-S6K, Akt, S6K, and glyceraldehyde 3-phosphate dehydrogenase (GAPDH) were purchased from Abcam. Goat anti-rabbit and anti-mouse IgG-HRP secondary antibodies were purchased from ABclonal Technology.

### Wound healing assay

Equal numbers of NSCLC cells were seeded in 6-well plates and cultured until they reached 95% confluency. A cell-free area with a width of approximately 1 mm was generated by scratching the surface with a pipette tip. The cells were washed twice with PBS and cultured in Dulbecco’s modified Eagle medium (DMEM) containing the indicated concentrations of STF. Images were captured using a microscope after 48 h of culture at 37°C. The wound healing ratio was calculated using ImageJ software (National Institute of Health, Bethesda, MA, United States).

### Tumor xenograft experiments

Tumor xenograft experiments were performed according to a protocol approved by the Institutional Animal Care and Use Committee of the Hefei Institutes of Physical Science, Chinese Academy of Science (Permit Number: DWLL-2020-48). BALB/c-nude mice, 6 weeks of age, were injected subcutaneously in the right posterior limb with a 100 μl suspension of 2 × 10^6^ A549 cells in an equal volume of Matrigel (BD Biosciences, Franklin, NJ, United States). Once the tumor volumes reached approximately 100–200 mm^3^, the mice were divided randomly into three groups (vehicle group: 150 μl of DMSO was dissolved in 2.85 ml of saline, and 100 μl was injected into each mouse; STF group: 200 mg/kg; cisplatin group: 5 mg/kg, purchased from Meilun Biotech, China). The mice were injected intraperitoneally with DMSO or STF emulsified in DMSO (the final concentration of DMSO was 5%) once a day and cisplatin twice a week for 23 days. The tumor volume and weight were measured. Hematoxylin-eosin (H&E) staining and immunohistochemistry (IHC) were also performed to estimate the expression of relevant proteins.

### Statistical analysis

Statistical analyses of our data were performed as previously described (Xu et al., 2019).

## Results

### Sheep tail fat contains high levels of C17:0 and suppresses non-small-cell lung cancer cell viability

**FIGURE 1:**
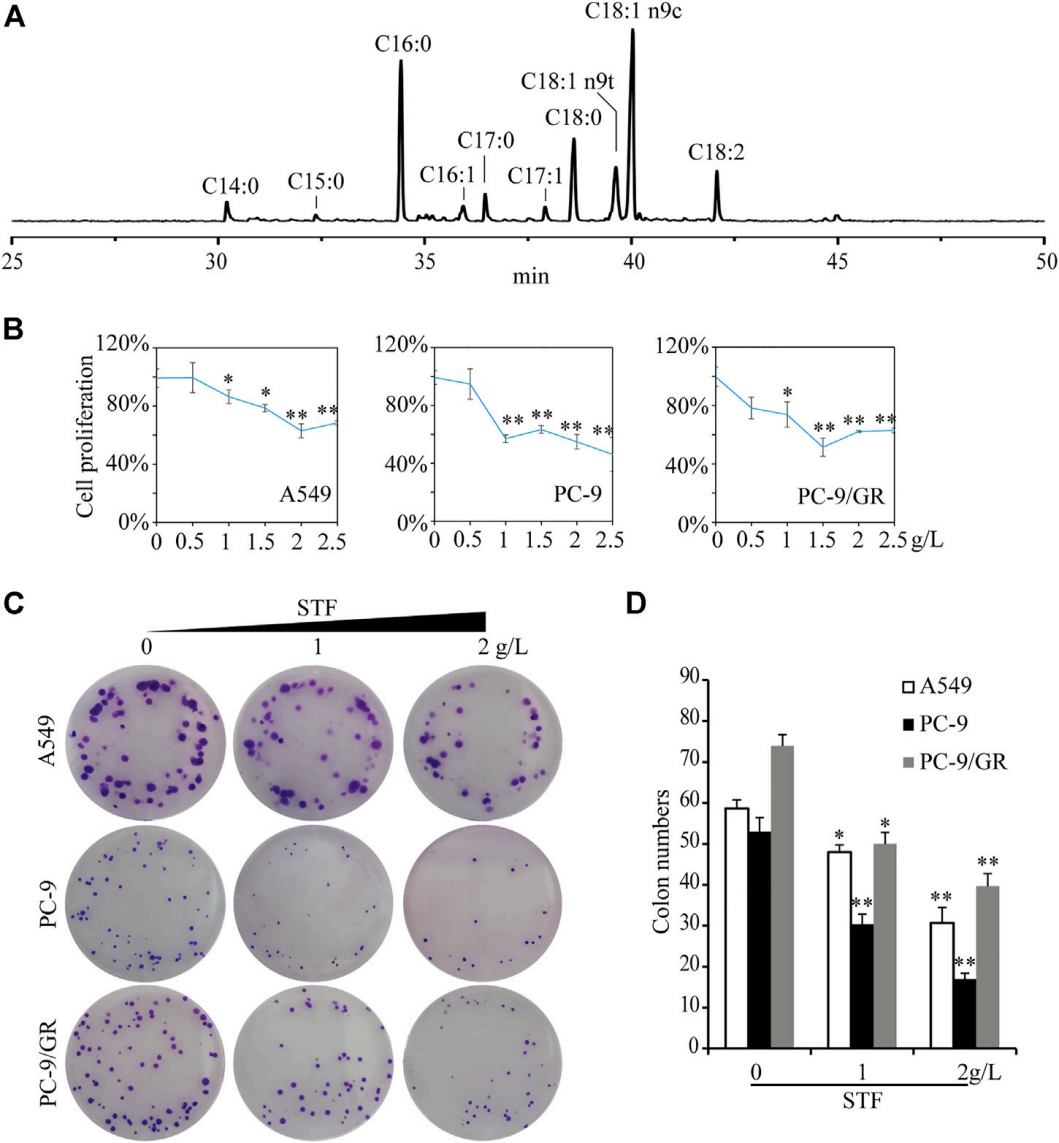
Inhibition of non-small-cell lung cancer (NSCLC) cell proliferation and colony formation by sheep tail fat (STF). **(A)** The fatty acid profile of STF was estimated by GC-MS analysis. **(B)** A549, PC-9, and PC-9/GR cells were plated overnight in 96-well plates. The cells were switched to a serum-free medium for 12 h, followed by treatment with different concentrations of STF for 48 h. The cell proliferation was determined using the 3-(4,5-dimethylthiahiazo-2-yl)-3,5-diphenyltetrazolium bromide (MTT) assay. **(C)** Three NSCLC cell lines were subjected to STF treatment for 2 weeks, and the formed colonies were imaged. **(D)** The number of colonies in each group was then counted. *p < 0.05, **p < 0.01.

STF and the other lipids were methylated and analyzed using GC-MS. As shown in Figure 1A and Table S1, palmitic acid (C16:0), stearic acid (C18:0), oleic acid (C18:1), and linoleic acid (C18:2) accounted for most of the fatty acids in common dietary lipids. Meanwhile, C17:0 made up approximately 3% of the total fatty acid profile in STF, and the C17:0 level in STF was found to be the highest of the edible lipids.

We then directly investigated the activity of STF against NSCLC cells *in vitro*. The concentration range of STF was based on its solubility in DMSO and the safe concentration of DMSO in our experimental cells and other reports (Batirel et al., 2018). As expected, the results of the MTT assay showed that STF administration reduced the proliferation rate of the three tested NSCLC cell lines in a dose-dependent manner (Figure 1B). The cell colony numbers for the three NSCLC cell lines were also significantly decreased in response to STF treatment (Figures 1C,D). In contrast, other common dietary lipids, apart from fish oil, did not suppress NSCLC cell proliferation. Furthermore, STF exhibited the greatest inhibitory effect against NSCLC cells among these lipids, including those extracted from other parts of sheep (Supplementary Figure S1). Among the animal lipids, beef tallow could not be subjected to this test because of its high melting point and limited solubility in DMSO. As shown in Supplementary Table S1 and Supplementary Figure S1, we also observed that lard shared a similar fatty acid composition as STF, except for C17:0. However, our results show that lard did not exhibit a similar inhibitory effect on NSCLC cells, indicating that the lack of an inhibitory effect with lard is due to the absence of C17:0. Conversely, C18:1-rich oils, such as olive and peanut oil, exerted a pro-proliferative effect. These results indicate that STF is unique in its ability to suppress the growth of NSCLC cells.

### Sheep tail fat induces reactive oxygen species accumulation and apoptosis in non-small-cell lung cancer cells

**FIGURE 2:**
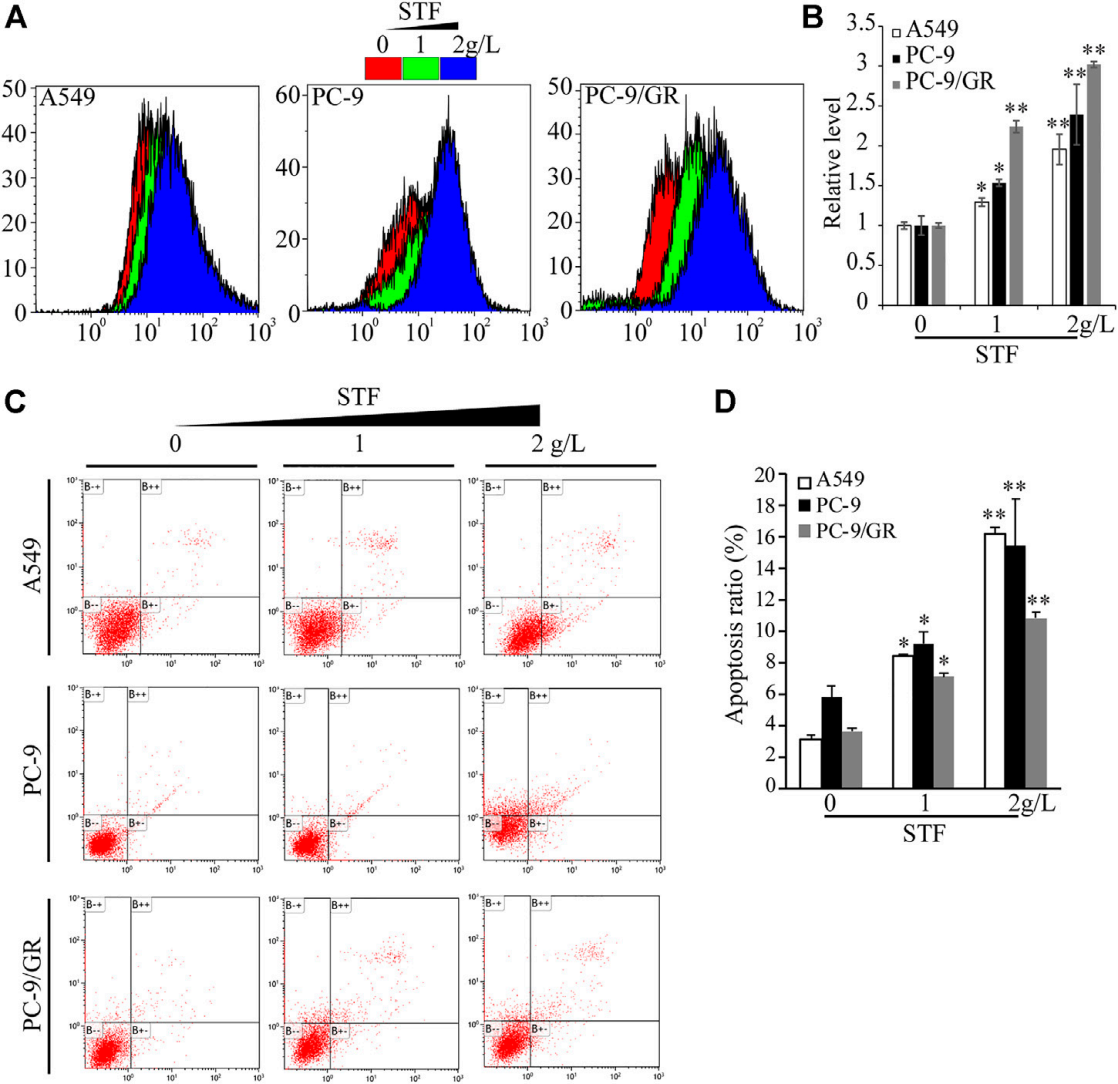
Exposure to STF triggered reactive oxygen species (ROS) production and NSCLC cell death. As described in Figure 1B, NSCLC cells treated with or without STF were subjected to measurement of ROS levels by flow cytometry **(A,B)** or stained with Annexin V-fluorescein isothiocyanate/propidium iodide (FITC/PI), followed by flow cytometry-based analysis to estimate the number of apoptotic cells **(C,D)**. *p < 0.05, **p < 0.01.

Next, we investigated the mechanism by which STF inhibits NSCLC cell proliferation. Antitumor compounds can inhibit cancer cell proliferation by inducing apoptosis. Generally, the intake of edible lipids can disrupt the metabolic rate and mitochondrial function of tumor cells, thereby inducing intracellular ROS production and apoptosis (Indran et al., 2011; Pavithra et al., 2018). Using DCFH-DA as a redox probe, we found that STF administration triggered the accumulation of intracellular ROS in a dose-dependent manner (Figures 2A,B). Cell apoptosis was then assessed by flow cytometry, which demonstrated that STF increased the Annexin V and PI-positive cell ratio in the NSCLC cells compared to that in the control group (Figures 2C,D). Collectively, our data suggest that STF administration induced the accumulation of intracellular ROS and subsequent apoptosis of these cells.

### Sheep tail fat inhibits non-small-cell lung cancer cell migration

**FIGURE 3:**
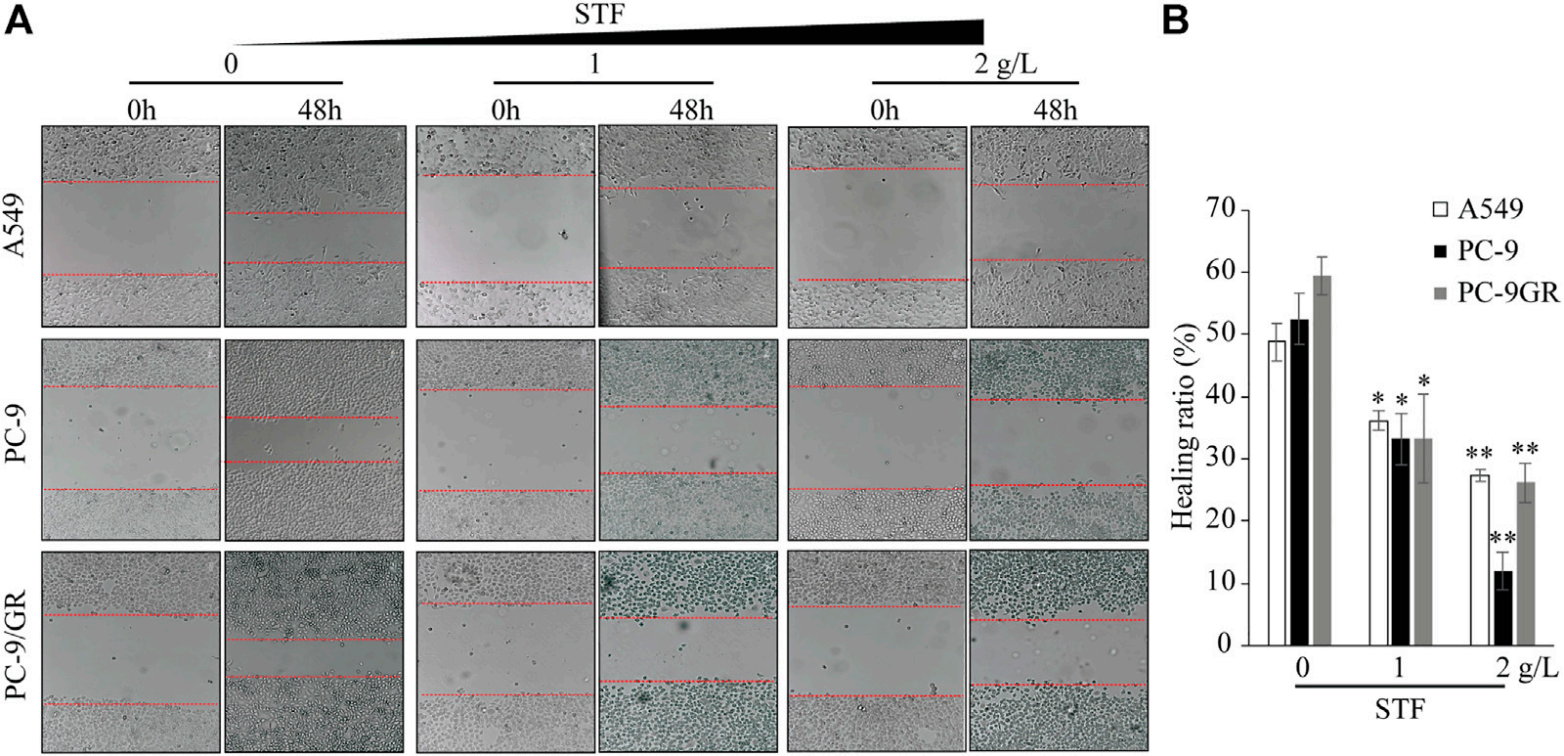
STF inhibited the wound healing capacity of NSCLC cells. A549, PC-9, and PC-9/GR cells were plated overnight. A cell-free area was generated with a pipette tip, and a fresh medium containing different concentrations of STF was added for 48 h. Cell images of the cell-free gap were captured **(A)** and analyzed statistically **(B)**. *p < 0.05, **p < 0.01.

Cell migration is another key property of cancer cells. Thus, the wound healing capacity of NSCLC cells was evaluated in response to STF treatment. As shown in Figure 3, compared to the control group, STF reduced the cell migration to the cell-free area in a dose-dependent manner. These results show that STF treatment suppressed the migration of NSCLC cells.

### Sheep tail fat downregulates the phosphoinositide 3-kinase/Akt signaling pathway and glucose transporter 1 levels

**FIGURE 4:**
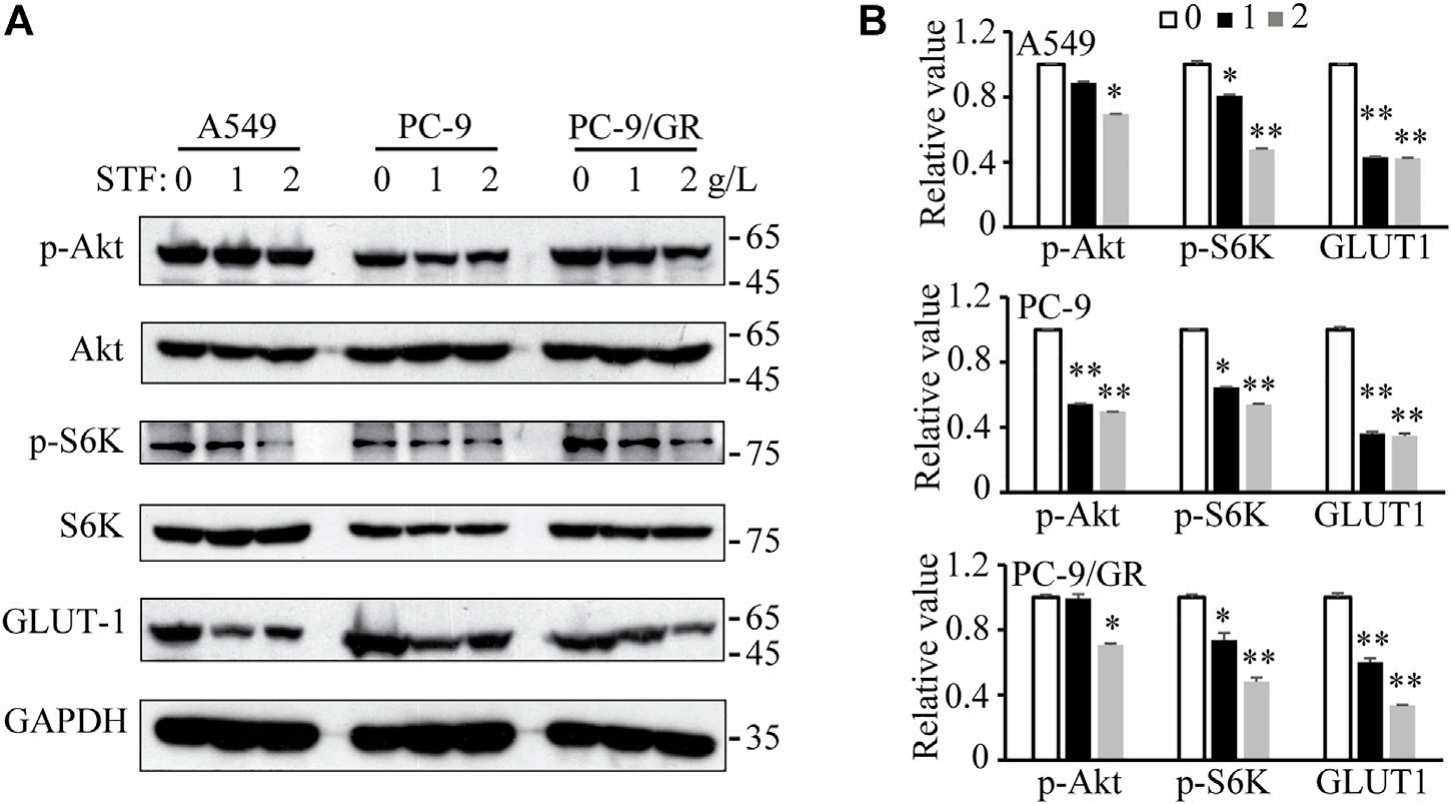
STF downregulated the PI3K/Akt signaling pathway and glucose transporter 1 (GLUT1) levels in NSCLC cells **(A)** Similar to Figure 1B, the cells were treated with STF and then subjected to immunoblotting to determine the levels of p-Akt, p-S6K and GLUT1. **(B)** The relative levels of these proteins are presented. *p < 0.05, **p < 0.01.

The uptake of SFAs may decrease membrane fluidity, further affect the functional activity of membrane and membrane protein dynamics. Among these, glucose uptake is one key membrane activity harnessed by tumor cells to fuel cell proliferation and invasiveness (Gatenby and Gillies, 2004; Ancey et al., 2018; Zambrano et al., 2019). In addition, the phosphoinositide 3-kinase (PI3K)/Akt signaling pathway is essential for NSCLC cell proliferation and tumor development (Tan, 2020) Also, PI3K/Akt signaling was reported to master the expression and location of GLUT1 (Wieman et al., 2007). Therefore, SFA addition or STF administration may alter membrane fluidity and glucose uptake through GLUT family, subsequently triggering the activation of PI3K/Akt signaling to accelerate NSCLC cell proliferation. To confirm this hypothesis, the expression of relevant proteins in the PI3K/Akt signaling pathway and glucose intake in NSCLC cells were determined. As shown in Figures 4A,B, immunoblotting revealed that STF administration reduced the levels of phosphorylated Akt and S6K in the cells. In addition, our results show that STF also decreased the GLUT1 levels, a critical protein that transports glucose into cells for ATP production (Gatenby and Gillies, 2004). Therefore, our data suggest that STF downregulated the PI3K/Akt signaling pathway and glucose intake in NSCLC cells, consistent with the function of C17:0 in our previous study (Xu et al., 2019).

### Sheep tail fat administration inhibits non-small-cell lung cancer *in vivo*

**FIGURE 5:**
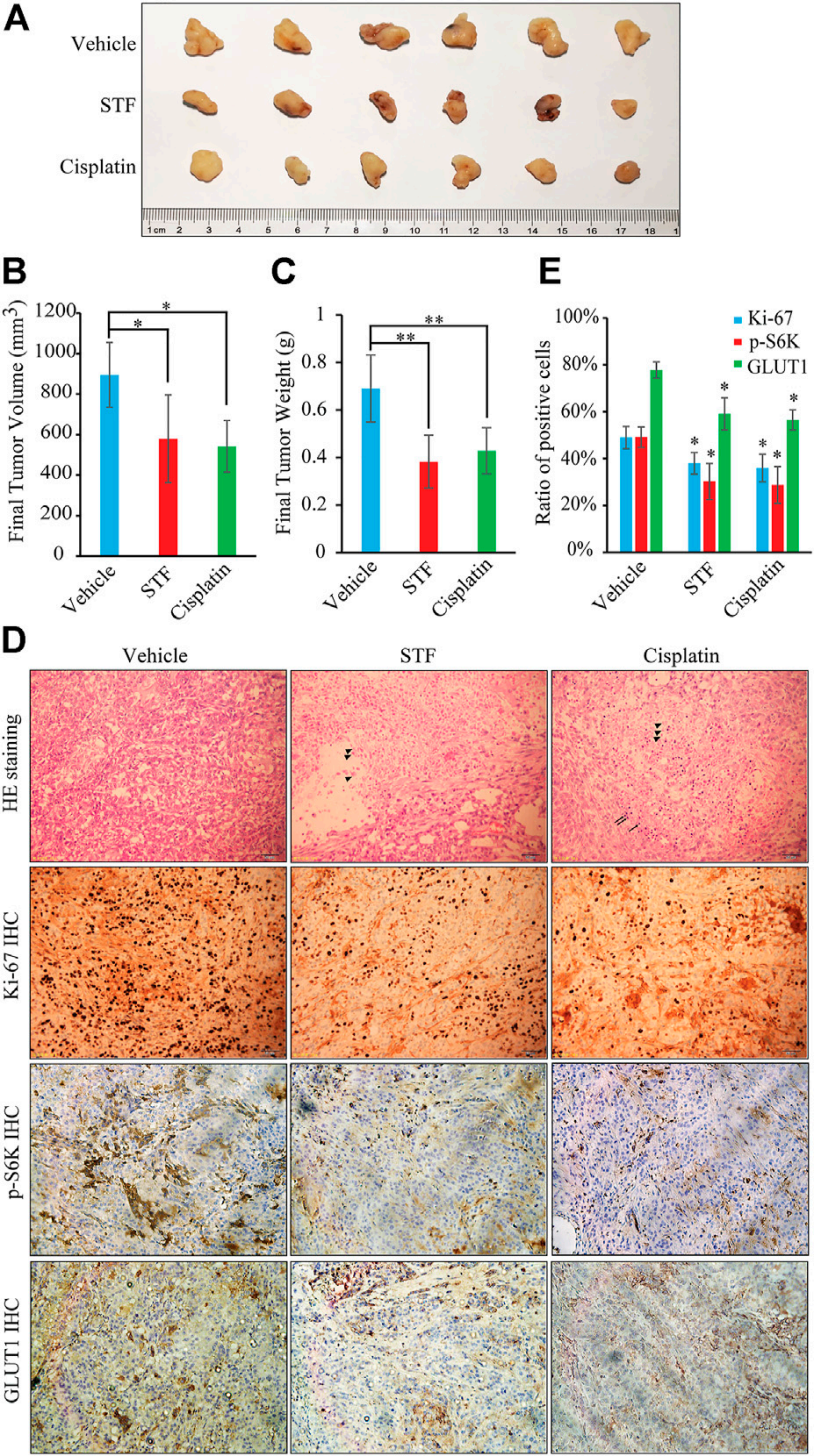
STF inhibited *in vivo* NSCLC tumor growth. **(A)** The antitumor activity of STF was evaluated in A549 cell-xenografted nude mice. The tumors were isolated by dissection and then photographed. **(B,C)** The final tumor volumes **(B)** and tumor weights **(C)** in each group were determined. **(D)** The hematoxylin-eosin (H&E) and immunohistochemistry (IHC) staining in each group was imaged using a microscope. The scale bar represents 50 μm. **(E)** The ratio of Ki-67, p-S6K, and GLUT1 positive cells in each group was compared statistically. Triangle: necrotic cells; Arrow: inflammatory cells. *p < 0.05, **p < 0.01.

We also investigated the antitumor effects of STF in a mouse tumor model. Nude mice transplanted with A549 cells were administered STF or dimethyl sulfoxide (DMSO) as vehicle control. The commonly used clinical cancer drug cisplatin was used as a positive control (Li et al., 2018). As shown in Figures 5A–C, STF inhibited tumor growth and reduced the final tumor weight relative to the DMSO control. Moreover, necrotic cells, a pathological phenomenon, were detected following H&E staining of the tumors of both the STF- and cisplatin-treated groups (Figure 5D). The IHC images revealed that the ratio of Ki-67, p-S6K, or GLUT1 positive cells in the STF and the cisplatin groups was much lower than that in the vehicle group, indicating that proliferation of the cancer cells was suppressed in response to treatment with either STF or cisplatin (Figures 5D,E). Collectively, our *in vivo* results suggest that STF exhibits antitumor activity in an A549 xenograft mouse model.

## Discussion

Edible oils extracted from plants and deep-sea fish are thought to be beneficial for human health because of their high content of UFAs (Wang et al., 2016). In contrast, common animal lipids are considered unhealthy due to their abundance of SFAs. Excessive intake of SFAs is considered to drive the occurrence and development of several health conditions, including obesity, hypertension, and cardiovascular diseases (Eckel et al., 2014; Kromhout et al., 2016; Binns et al., 2017). However, other studies have found that SFAs are not the underlying cause of these conditions (Malhotra, 2013; Chowdhury et al., 2014; Li et al., 2015; Dehghan et al., 2017; Malhotra et al., 2017; Veum et al., 2017). It was recently reported that SFAs also play important roles in various diseases. For instance, intake of SFAs can reduce the severity of pancreatitis in humans (Khatua et al., 2021). C16:0 decreases the metastatic capacity of hepatocellular carcinoma cells (Lin et al., 2017). These reports suggest that animal lipids rich in certain SFAs may enhance the efficacy of clinical therapies for various diseases. As a functional OCSFA, C17:0 intake has been inversely correlated with multiple diseases (Holman et al., 1989; Khaw et al., 2012; Meikle et al., 2013). Here, we found that, among the common dietary lipids, C17: 0 is abundant in STF. Interestingly, our results show that STF is unique in its ability to inhibit NSCLC cell proliferation compared to other common dietary lipids.

Different edible lipids may have different effects on NSCLC cells. In our study, C18:1-rich edible oils promoted the proliferation of NSCLC cells in a manner similar to that observed with C18:1 treatment alone (Supplementary Figures S1, S2). However, lipids containing high levels of SFAs (particularly C17:0) and low levels of UFAs (C18:1) may exhibit a special inhibitory effect against NSCLC cells (Supplementary Figure S1), consistent with a recent report that showed that an increased ratio of UFA/SFA triggered by stearoyl-CoA desaturase 1 (SCD1) could reduce the cytotoxicity of SFA toward tumor cells (Lien et al., 2021). These results also suggest that certain SFAs, or other entities, contributed to the inhibition by counteracting the action of C18:1. Furthermore, we found that three SFAs, namely C16:0, C17:0, and C18:0, exerted a much more potent inhibitory effect on NSCLC cells, of which C17:0 had the lowest IC_50_ value (Supplementary Figure S3A). Accordingly, the concentration of C17:0 here was approximately 0.07 g/l in 2.5 g/l STF, and the IC_50_ values of C17:0 in our previous study were approximately 0.02 and 0.04 g/l for PC-9 and PC-9/GR cells, respectively, indicating that C17:0 in STF may underlie the similar inhibitory effect (Xu et al., 2019). Also, the comparison between C17:0, STF, and cisplatin was performed in Supplementary Figure S4. C17:0 also exhibited a strong inhibitory effect against C18:1-treated NSCLC cells (Supplementary Figure S3B). C16:0 and C18:0 also had pronounced inhibitory effects on NSCLC cells (Supplementary Figure S3A), which is consistent with other reports (Carrillo et al., 2012; Lin et al., 2017). Therefore, three fatty acids (C16:0, C17:0, and C18:0) may underlie much of the activity of STF. However, the roles of C16:0 and C18:0 appear to be controversial when comparing the effects of STF and lard (Supplementary Figure S1). Thus, C17:0 may be the most representative contributor to the activity of STF. Thus, more in-depth experiments referring to the contributors will need to be performed in the future.

The antitumor potential of STF was further confirmed in our xenograft mouse model. Our results show that STF exhibited antitumor activity in A549 cell-xenografted mice, as evidenced by the slower tumor growth and the reduced final tumor weight, as well as the results of the H&E staining and IHC staining (Figure 5). Cisplatin is one of first-line drug for the treatment of NSCLC and its inhibitory effect is much better than that of STF *in vitro* (Supplementary Figure S4). However, its *in vivo* results are similar to that of STF here. During the process, we found that the mouse weight in cisplatin group was much lower than that in STF group (Supplementary Figure S5). Therefore, cisplatin treatment may be toxic due to its side-effect to normal cells, subsequently dampen its *in vivo* effect. Interestingly, an epidemiologic report has shown that the incidence of lung carcinoma in the Uygur population is much lower than that in the Han population (6.2% vs. 93.8% out of a total of 2,047 cases) in Urumqi, Xinjiang Autonomous Region, China between 2003 and 2012, where the population of Uygur and Han accounted for 46.62% and 38.99%, respectively (Ma et al., 2014). Differences in habits, such as the intake of STF, may partly contribute to the difference in the incidence of lung cancer. In addition, targeted metabolomics analysis has reported that C17:0, one of the top five most significant metabolites, was much lower in patients with lung cancer than in the healthy group (Qi et al., 2021). These reports suggest that supplementation with C17:0-rich STF might be beneficial for lung cancer therapy. Nevertheless, more evidence from *in vivo* studies with long-term intake of STF is needed to elucidate its role in NSCLC.

In our animal experiment, 200 mg/kg of STF was selected to treat mouse for over 20 days. In fact, 400 mg/kg group was also designed and both the two groups showed similar inhibitory effect on tumor growth, while did not alter mouse weights (Supplementary Figure S6). Other lower concentrations were not performed and this is a limitation in our study. The dose of 200 mg/kg was a commonly used concentration for potential lipids in antitumor experiments. According to the normalization method of body surface weight area (BSA) (Reagan-Shaw et al., 2008), this dose also belongs to the normal range of meaningful concentration of the extract for pharmacology experiments and other reports (Murphy et al., 2011; Cockbain et al., 2012; Li et al., 2018; Nanda et al., 2019; Heinrich et al., 2020; Kleckner et al., 2021). Although our results suggested that STF could safely suppress tumor cell growth in xenograft model, more further investigations should be performed in the future study, such as the lethal dose of STF and its combination effect with clinical drugs.

Although C17:0 increases with animal age in sheep (Watkins and Frank, 2019), the concentration of C17:0 is still much lower in STF than that of other fatty acids, such as C18:0. To assess the role of high levels of C17:0, fatty acid mixtures with different ratios were generated. The results shown in Supplementary Figure S7 indicate that enrichment of C17:0 may result in a more significant antitumor effect of fatty acid mixtures. However, little is known about C17:0 enrichment in ruminant lipids. Therefore, optimization of C17:0 in lipids and its antitumor potential against lung cancer cells require further investigation.

To our knowledge, this is the first study to investigate the role of STF on cancer cells. We found that STF has a relatively high abundance of C17:0 and was unique in its pronounced inhibitory effect on NSCLC cell proliferation. These results favor the application of STF as adjuvant therapy for NSCLC.

## Data Availability

The original contributions presented in the study are included in the article/Supplementary Material, further inquiries can be directed to the corresponding authors.
