# Supplementary material for: Sheep tail fat inhibits the proliferation of non-small-cell lung cancer cells in vitro and in vivo
Source: Front Pharmacol. 2022 Aug 11;13:917513. doi: 10.3389/fphar.2022.917513 (PMC9403308; doi:10.3389/fphar.2022.917513)
Supplement: Supplementary file 1 [file DataSheet1.docx]

Supplementary Material

# Supplementary Figures and Tables

## Supplementary Figure 1


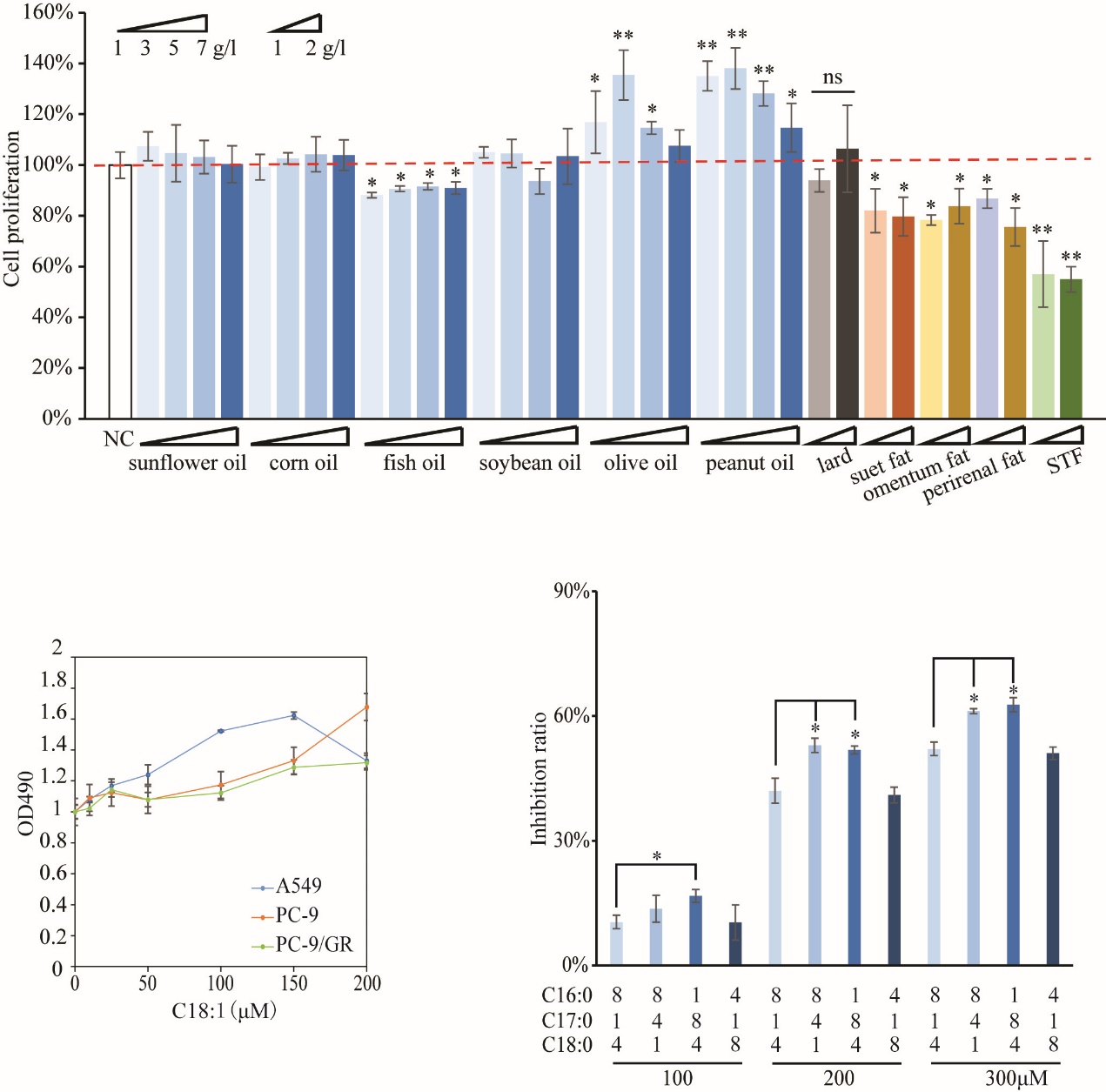


**Supplementary Figure 1.** The impact of common edible lipids on NSCLC cells. Equal amounts of PC-9 cells were subjected to the treatment of common edible oils with varying concentrations. The MTT results were acquired through the detecting of absorption at 490 nm. **p*<0.05, ***p*<0.01, ns: no significance.

## Supplementary Figure 2


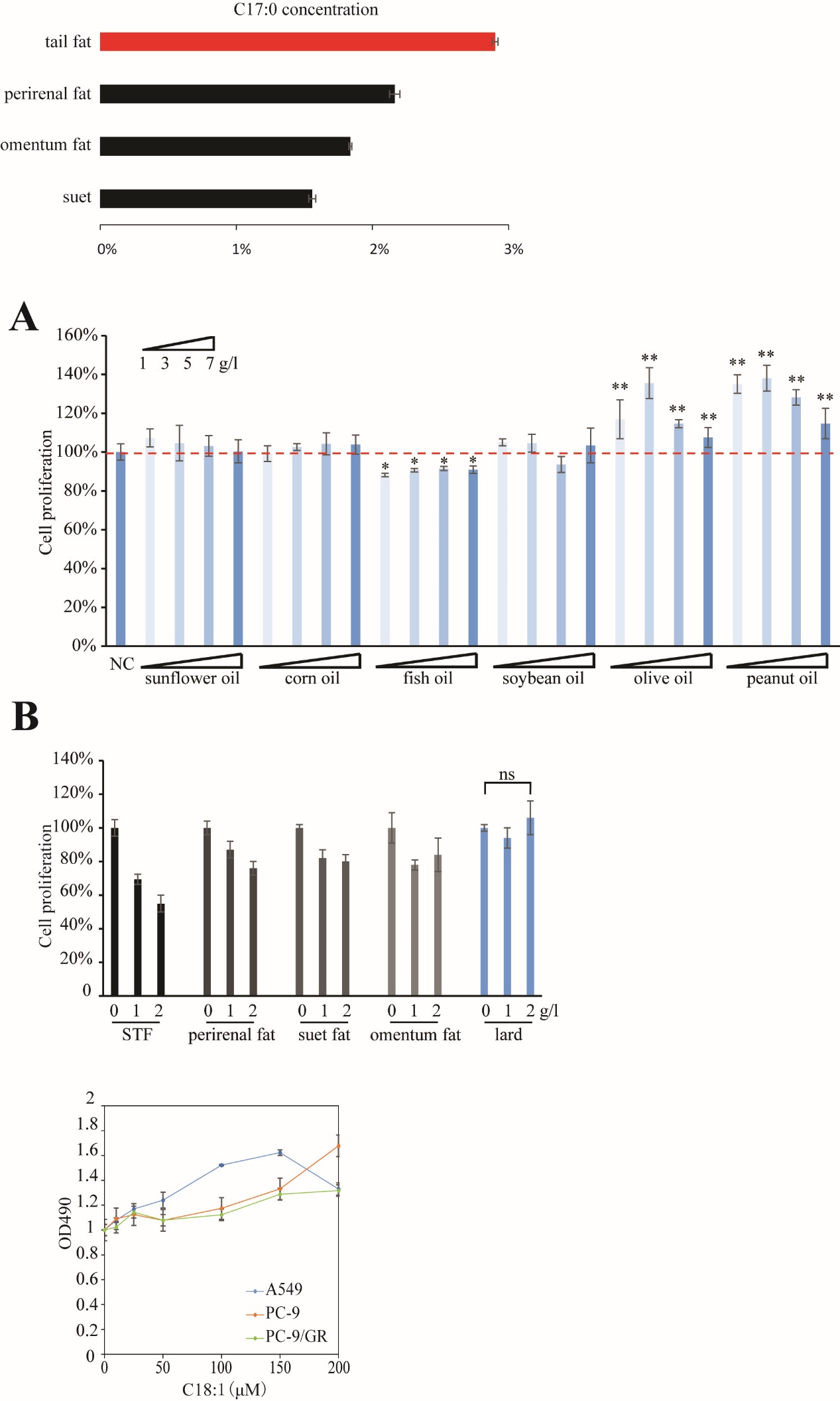


**Supplementary Figure 2.** The impact of C18:1 on NSCLC cells. Similar to Figure S1, three NSCLC cells were subjected to the treatment of C18:1 and the absorption was measured.

## Supplementary Figure 3


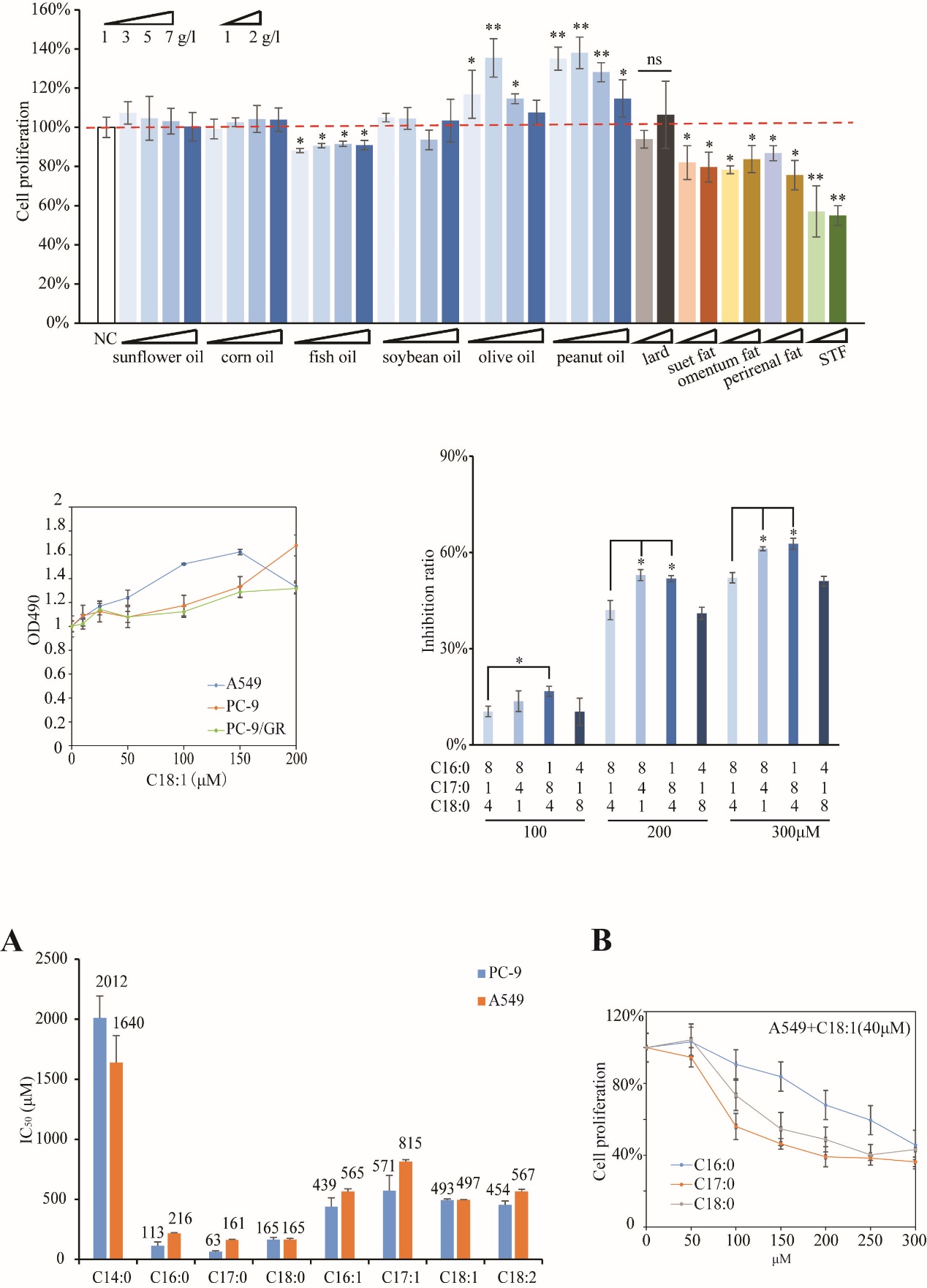


**Supplementary Figure 3.** The inhibitory effect between FAs and the counteraction to C18:1. (**A**) The IC_50_ values of most fatty acids in STF. (**B**) A549 cells were subjected to the treatment of C18:1 (40 μM) and gradient concentration of C16:0, C17:0 or C18:0, and cell proliferation was obtained.

## Supplementary Figure 4


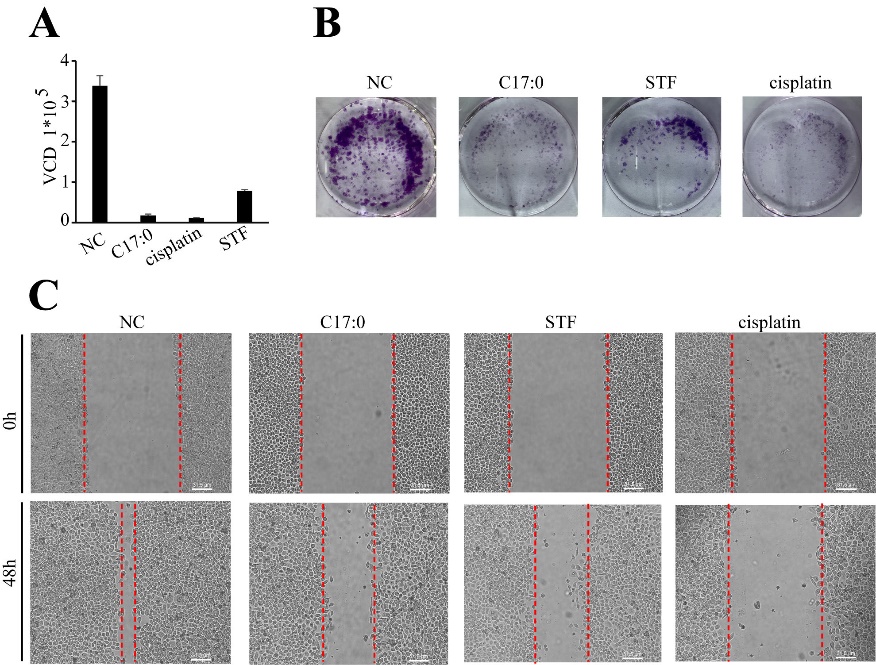


**Supplementary Figure 4.** The *in vitro* inhibitory comparison between STF, C17:0, and cisplatin. PC-9 cells were equally seeded in 6-well plates overnight. C17:0 (100 μM), STF (2 g/L), or cisplatin (2.5 μg/mL) were added. Cell growth (A), colony formation (B), and cell migration (C) were obtained according to the relevant methods in the manuscript.

## Supplementary Figure 5

**Supplementary Figure 5.** The mouse weight during the treatment. As described in Figure 5, the mouse weight in each group was measured.

## Supplementary Figure 6


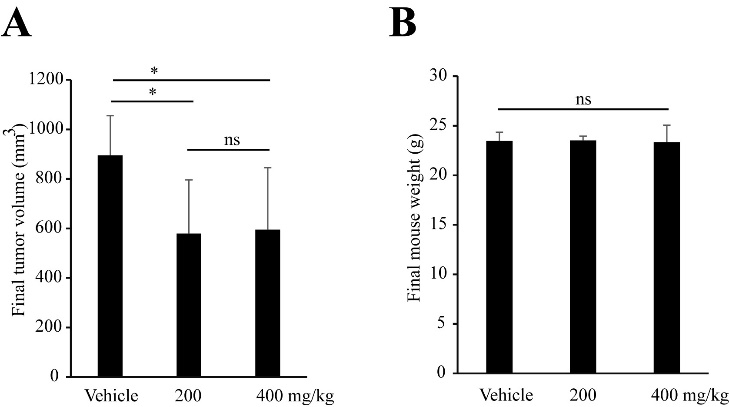


**Supplementary Figure 6.** The comparison of final tumor volume and mouse weight between 200 mg/kg and 400 mg/kg groups. **p*<0.05. ns: no significance.

## Supplementary Figure 7


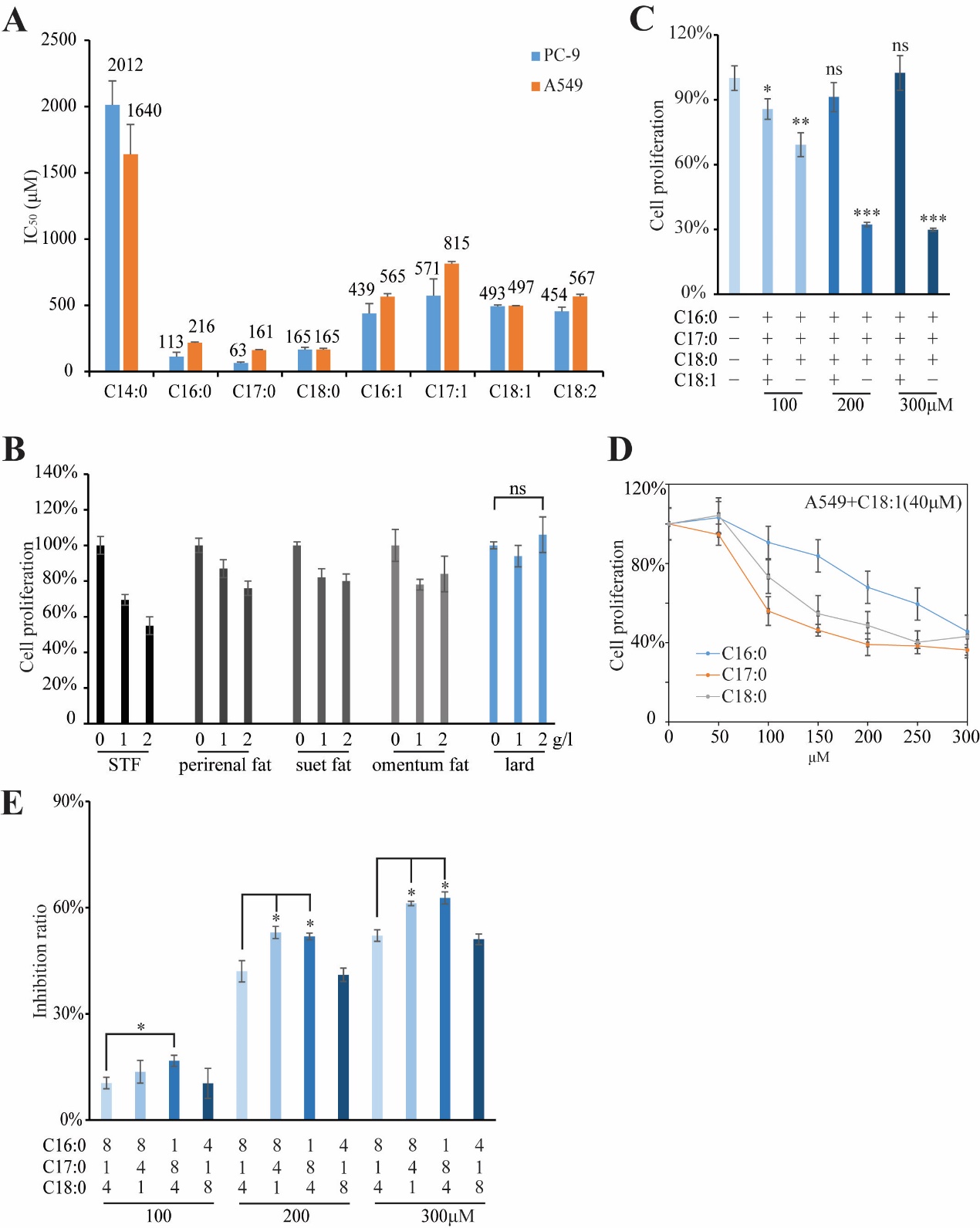


**Supplementary Figure 7.** The impact of fatty acid mixtures with different C17:0 levels on NSCLC cells. The fatty acid mixtures with similar ratios in STF (the ratio of C16:0/C17:0/C18:0 = 8/1/4), or other ratios were added into PC-9 cells with different concentrations. Similarly, the inhibition ratios were obtained through the MTT method. **p*<0.05.

**Supplementary Table 1**. Fatty acid composition and percentage in STF and other dietary lipids

| fatty acid | STF^1^ | Sheep perirenal fat | Sheep omentum fat | Sheep suet | beef tallow | fish oil (1) | Lard (2) | sunflower oil (3) | corn oil (3) | soybean oil (2) | olive oil (4) | oleic-rich peanut oil (5) |
| --- | --- | --- | --- | --- | --- | --- | --- | --- | --- | --- | --- | --- |
| C6:0 | - | - | - | - | - | - | 1.91±0.07 | - | - | - | - | - |
| C14:0 | 2.60±0.02 | 2.51±0.02 | 1.56±0.01 | 3.19±0.02 | 2.58±0.01 | 2.41±0.09 | 1.19±0.07 | - | - | 0.08±0.05 | - | - |
| C15:0 | 0.97±0.07 | 0.65±0.02 | 0.65±0.01 | 0.65±0.01 | 0.49±0.00 | 0.23±0.02 | - | - | - | - | - | - |
| C16:0 | 23.63±0.21 | 24.19±0.04 | 20.29±0.05 | 26.64±0.05 | 24.81±0.06 | 23.65±0.12 | 24.77±0.20 | 09.28±0.03 | 13.98±0.07 | 10.84±0.38 | 11.02±0.01 | 6.01 |
| C16:1 | 2.03±0.04 | 2.17±0.05 | 2.28±0.06 | 2.12±0.01 | 1.89±0.01 | 9.31±0.10 | 1.76±0.07 | - | - | 0.08±0.08 | - | - |
| C17:0 | 3.02±0.05 | 2.17±0.00 | 1.84±0.00 | 1.56±0.00 | 1.46±0.00 | 0.23±0.02 | - | - | - | - | - | - |
| C17:1 | 1.29±0.03 | 0.55±0.02 | 0.69±0.02 | 0.42±0.00 | 0.37±0.00 | - | - | - | - | - | - | - |
| C18:0 | 13.02±0.13 | 27.10±0.16 | 27.24±0.02 | 29.54±0.03 | 28.65±0.08 | 6.21±0.08 | 13.52±0.14 | 04.03±0.01 | 01.73±0.02 | 4.31±0.08 | 3.73±0.01 | 3.13 |
| C18:1 | 44.61±0.10 | 34.48±0.03 | 36.85±0.02 | 29.38±0.05 | 34.80±0.02 | 31.03±0.10 | 42.37±0.66 | 23.71±0.02 | 28.13±0.06 | 23.92±0.37 | 78.08±0.02 | 81.84 |
| C18:2 | 4.60±0.15 | 1.54±0.07 | 1.71±0.03 | 3.10±0.04 | 3.72±0.14 | 12.21±0.07 | 12.48±0.13 | 65.00±0.05 | 52.89±0.16 | 53.35±0.30 | 4.95±0.00 | 4.10 |
| C18:3 | 0.18±0.04 | 0.19±0.02 | 0.08±0.01 | 0.11±0.01 | 0.19±0.01 | 1.35±0.02 | 0.52±0.29 | - | 00.79±0.01 | 5.97±0.08 | 0.63±0.01 | 01.00±0.02 |
| C19:0 | 0.17±0.04 | 0.30±0.01 | 0.28±0.01 | 0.10±0.01 | 0.24±0.01 | - | - | - | - | - | - | - |
| C20:0 | - | - | - | - | - | - | - | - | - | - | 0.45±0.01 | 1.00 |
| C20:1 | - | - | - | - | - | 1.08±0.06 | - | - | - | - | 0.25±0.00 | 1.39 |
| C20:2 | - | - | - | - | - | 0.21±0.01 | - | - | - | - | - | - |
| C20:4 | - | - | - | - | - | 0.39±0.03 | - | - | - | - | - | - |
| C20:5 | - | - | - | - | - | 4.48±0.03 | - | - | - | - | - | - |
| C22:0 | - | - | - | - | - | - | - | - | - | - | - | 2.54 |
| C22:6 | - | - | - | - | - | 7.21±0.12 | - | - | - | - | - | - |
| C23:0 | - | - | - | - | - | - | - | - | - | - | 0.89±0.00 | - |

^1^sheep tail fat

# Reference

1. Zhao T., Ying X., Zhang B., Deng S., Ma L., Zhu L., et al. (2021). Preparation and physicochemical properties of *Larimichthys Crocea* fish oil. *China Oils Fats* 46(10), 6-10. [in Chinese]. doi: 10.19902 /j.cnki.zgyz.1003-7969.2021.03.101.

2. Gao J., Ma T., Fang Q., Qin X., Wang K., Yang X., et al. (2017). Study on compatibility of binary mixture systems between lard oil and three kinds of plant oil. *Sci. Technology. Food Industry* 38(13), 53-57. [in Chinese]. doi: 10.13386/j.issn1002-0306.2017.13.010.

3. Zhang W., Li H., Zhang Z., Li H., Cui L., Chen T. (2021). Fatty acid composition and antioxidant properties of eight different vegetable oils. *China Oils Fats* 46(4), 68-71. [in Chinese]. doi: 10.19902 /j.cnki.zgyz.1003-7969.2021.04.014.

4. Luo X., Gao P., Hu C., and He D. (2022). Comparative study on Extra Virgin olive oil imported from Spain and Italy. *China Oils Fats.* 47(06), 46-52. doi: 10.19902/j.cnki.zgyz.1003-7969.210530.

5. Zheng C., Yan M., Zhou Q., Huang F., Deng Q., Guo P., et al. (2014). Contents of fatty acid and minor component and oxidative stability of high-oleic peanut oil and normal-oleic peanut oil. *China Oils Fats* 39(11), 40-43. [in Chinese].
